# Sense of Place and Perceived Impacts in the Rural Industrialized Nexus: Insights for Sustainability Pathways

**DOI:** 10.1007/s00267-024-01969-3

**Published:** 2024-04-05

**Authors:** Deseret Weeks, Jeffrey Jenkins

**Affiliations:** Department of Management of Complex Systems, University of California, Merced, CA USA

**Keywords:** Sustainability, Water-Energy-Food Nexus, Sense of Place, Rural Industrial Development

## Abstract

As representative of the water-energy-food nexus, fossil fuel development and industrial agriculture are rural industries that continue to expand and increasingly occur in the same areas. Being a top agricultural export county and the fossil fuel capital of California while ranking among the worst in the US for industrial pollution, Kern County is a poster child of rural nexus development and, thus, an essential place for initiating sustainability transitions. Such transitions rely on policy support and the adoption of methods by individuals and communities who may disagree with such changes. While sense of place and impact perceptions are recognized as playing critical roles in sustainability management, they have yet to be utilized in nexus research. A survey (*N* = 256) of the perceived impacts of nexus industries with place meaning and place attachment as possible drivers for perceptions was conducted in nexus industry pollution exposure risk zones. Factor analysis and bivariate correlations showed that place meaning and place attachment are drivers for perceptions while also being drivers for concern for changes in nexus industries. While perceptions of impacts indicated contested place meanings, participants strongly perceive the economy and environment as being in decline. To build support for sustainability policy, directing funds from Kern County’s renewable energy industry to local sectors of society, implementation of regenerative agriculture, cooperative management, and nurturing place meaning as aligned with nature’s restorative quality are important paths forward. These nexus management foci could strengthen place attachment, build trust in government, and repair environmental alienation.

## Introduction

While the water-energy-food (WEF) nexus has been adopted broadly for sustainability management, foci of its use and application as an analytical tool have been largely based on resource security to meet the demands of population and economic growth (Albrecht et al. 2018; Wiegleb and Bruns 2018; Artioli et al. 2017). These predominant goals and methods have stymied the needed focus on drivers of global change as part of WEF nexus processes, environmental injustice, and local livelihoods, among others (Liu et al. 2018; Albrecht et al. 2018; Biggs et al. 2015; Allouche et al. 2015). A growing consensus among sustainability scholars is that the natural sciences have dominated WEF nexus discourses and that, to align policy with sustainability needs, social scientific approaches need to be promoted (Allouche et al. 2015; Wiegleb and Bruns 2018). Accordingly, social science approaches provide for the ability to address issues of inequity, environmental justice, and systemic power as well as incorporate local knowledge, culture, and experience of management outcomes in science and policy (Wiegleb and Bruns 2018; Allouche et al. 2015; Haggerty et al. 2019). Further, decisions about policies that affect socio-environmental management can be improved by incorporating local perceptions and values, the understanding of which comes largely from social science approaches (Mulvaney et al. 2020; Craik 1973; Adger 2006; Dietz et al. 2005).

Across the social sciences, sense of place (SoP) has gained recognition as playing a pivotal role in sustainability management and transitions. However, it has yet to be utilized in WEF nexus research and management. As a social theory, SoP provides explanations and insights into human connections to and meanings of a place (Mulvaney et al. 2020). Tuan (1975) explains that “place is a center of meaning constructed by experience.” Accordingly, SoP has been identified as being a critical construct undergirding values and actions, thus possibly providing an essential mechanism for sustainability management of complex socio-environmental systems (Milligan 1998; Chapin III et al. 2012; Chapin III and Knapp 2015; Stedman 2016). For example, social constructs of sense of place represent meaning-making, or cognitive processes that often undergird place attachment, the latter of which has been attributed to conservation behavior (Lee 2011; Kyle and Chick 2007). Stedman (2016) suggests SoP, being systematically distributed through society via meaning-making processes, can provide a crucial mechanism for complex socio-ecological systems research and management to escape a theoretical rigidity trap that causes a tendency to overlook personal experience, human cognition of environmental problems, experience of issues, and local perspectives.

The utilization of SoP in sustainability-related research in the separate components of the rural industrialized WEF nexus provides evidence of its value. For example, Davenport and Anderson (2005) investigated SoP and perceptions of landscape change as related to the economic development of the Niobrara National Scenic River. They found river meanings as part of SoP undergirds place attachment, which can shape attitudes and behaviors about planning and management (Davenport and Anderson 2005). Mulvaney et al. (2020) call for researchers to use SoP as a “cultural ecosystem indicator,” considering that measuring the social value of water quality provides a pivotal link to biophysical indicators of water quality important for water quality restoration. Meanwhile, Jacquet and Stedman (2013) explore SoP as a driver for the perceptions of the impacts of wind energy versus fossil fuel development projects, underscoring the importance of perceptions in supporting or opposing such development projects in the face of climate change. Eaton et al. (2019) identify rural working landscapes as essential locations for conservation and advise using SoP as an empirical measure to capture the interdependent relations between the “social, economic, and environmental well-being experienced by farmers.” They explain that understanding how SoP “operates” in rural working landscapes can provide needed insight into motivation factors for conservation practices (Eaton et al. 2019).To promote social science approaches and successful sustainability management outcomes, this research explores place meaning, place attachment, and perceived impacts in the rural industrialized nexus. While the perceived impacts of industry remain an underexplored aspect of WEF nexus research and management, SoP and its role in perceptions of industrial development impacts has yet to be a focus in WEF nexus research and management. Meanwhile, perception of the impacts of industrialization and SoP has been found to be essential for sustainability management policy support. As development continues to follow the path towards industrialization and global demand for food and energy security continues to rely on fossil fuel development and industrial agriculture, understanding SoP and its role in impact perceptions will be important for sustainability transitions in the WEF nexus. This research thus seeks to ascertain the nature of place attachment, place meaning, and perceived impacts of industrialization, as well as correlations between SoP dimensions and perceived impacts in the rural industrialized WEF nexus. The research questions here are (1) What are the socio-environmental impacts, positive or negative, of rural WEF nexus industries perceived by residents, particularly as these industries intersect with water, and in what ways do aspects of place attachment and place meaning represent drivers of perception? (2) How might SoP in the WEF nexus be utilized to better achieve sustainability management and transition policy support? (3) How does the above differ between industrial agriculture and fossil fuel development?

This research centralizes on the rural-industrialized WEF nexus for key reasons. As representative of the WEF nexus, fossil fuel development and industrial agriculture are rural industries that increasingly occur in the same areas and continue to expand (Measham et al. 2016; Rockström et al. 2014). Meanwhile, the demands for energy and food within the broader market rationale of the global economic system are contradictory to ecological limits to this industrial growth, making the rural WEF nexus an essential focal point for sustainability transitions (Meadows et al. 1992; Vargas et al. 2023). For example, while industrial agriculture has been found to be a major driver of global change crises and trajectories towards planetary boundary exceedance, coupled economic-population growth trends provide for projections of 10^9^ hectares of natural ecosystems to be converted to industrial agricultural lands by 2050 (Tilman et al. 2001; Campbell et al. 2017). This conversion is expected to be accompanied by a 2^4^–2.^7^-fold increase in nitrogen and phosphorus-driven eutrophication and a similar increase in the use of pesticides (Tilman et al. 2001). Meanwhile, the continued reliance on fossil fuels for energy security and a multitude of other industries/products has been supported by a rapid expansion in unconventional oil and natural gas development (hydraulic fracking) (Black et al. 2021). While this expansion has provided income security, it has also led to the degradation of environmental quality and human health while also contributing to the climate crisis (Mayer 2016; Black et al. 2021). Moreover, Industrial agriculture and unconventional oil and gas development are notorious for water consumption and contributions of chemicals to local water resources (Shrestha et al. 2017; Khan and Hanjra 2009; Chittick and Srebotnjak 2017). While sustainability transitions for agriculture and energy production have been prescribed (i.e., decarbonization, regenerative agriculture), such transitions rely on policy support and the adoption of sustainability management methods by communities who may disagree with such changes (Escobar 2015; Shiva 2008; Lamine 2011).

## Place Attachment, Place Meaning, and Perceived Impacts

SoP, as a complex construct, comprises feelings, beliefs, meanings, symbols, and values developed through interactions and experiences of people within a setting (Chapin III et al. 2012). Classical development of SoP within human geography explored the sense of belonging as associated with sentiments tied to a setting and place, meanings and feelings associated with nurturing, stability, or interpretations of events or experiences, as well as values associated with family, culture, public institutions, and government policy (Tuan 1975). Tuan (1975) philosophized that the development of SoP depends on time, considering interactions with, involvement in, and experiences of a place take time to accrue. Building from these foundations, place attachment and place meaning are often identified as two key concepts of SoP, with place attachment being dependent on place meaning (Brehm et al. 2013). For example, Stedman (2008) explained that place attachment is a function of place meanings, which are made up of cognitions and personal or shared beliefs, yet that place meaning alone may be more relevant to environmental managers, considering conflicting views on management often stem from place meanings. Meanwhile, place attachment may be a driver of concern for environmental change (Jacquet and Stedman 2013). It is thus valuable to explore place attachment and place meaning together to gain a holistic picture of SoP.

Place meanings are cognitive and descriptive elements of attitudes about spatial settings (Brehm et al. 2013). Place meanings evolve through the experiences of an individual and the creation of memories (Quinn et al. 2018). Physical, experiential, and socially constructed aspects of a place are thus central to place meanings (Stedman 2003). The lived experience provides for the development of place meanings (Tuan 1975). Common meanings of a place among a group of people represent place meaning as being community, culturally, and economically relevant, which may shape attitudes and behaviors towards the environment (Davenport and Anderson 2005). Relatively, Cresswell (2008) noted that while sense of place as meanings, individual and shared, are associated with a place, temporal changes in sense of place are rooted in political economic dynamics of certain times and warned that “the sound of the beating heart of sense of place is getting lost behind corporate development.” Alternately, Stedman (2002) highlights that humans are willing to fight for places more central to their identities and perceived as being in less-than-optimal conditions. Thus, research must deal with perceptions, meanings, and beliefs people attribute to and have about a spatial setting (Davenport and Anderson 2005; Stedman 2002; Jenkins 2011).

Place attachment represents the strength of a connection or bond between an individual or group of people with a place which can be emotional, biological, cultural, familial, and/or economic (Stedman 2008; Brown et al. 2015; Hernández et al. 2007; Cross et al. 2011). Relatedly, two main dimensions of place attachment are place identity and place dependence. Proshansky (1978) defines place identity as “those dimensions of self that define the individual’s personal identity in relation to the physical environment by means of complex patterns of conscious and unconscious ideas, feelings, values, goals, preferences, skills, and behavior tendencies relevant to a specific environment.” Place dependence stems from relationships with a place and beliefs that a place satisfies psychological and/or physical needs (Davenport and Anderson 2005). While the strength of place attachment is most often correlated with length of residence, place meaning and identity also play important roles in the strength of people-place ties (Hernández et al. 2007). For example, social capital of community, family, and culture provide for emotional ties and personal identity, all of which have relations to places, attachment to those places, and concern for negative environmental change (Giuliani 2003; Brehm et al. 2013). Giuliani (2003) and others point out that conflicts in places can arise when there are disagreements between groups who have strong attachments to the same place while having different perceptions and values associated with place-based management (Chapin III and Knapp 2015; Jenkins 2018).

Clearly, place meaning and place attachment play important roles in human perception of a place, which is why SoP is often used in conjunction with perceived impacts in sustainability management research. For example, Davenport and Anderson (2005) ask, “What happens to sense of place when places change?” and “What happens when landscape change threatens place meanings and emotions?” They found that some residents opposed commercial development on the local river due to perceived threats to place meanings (identity, nature, tonic) associated with the river. However, some viewed the development as positive due to river meanings associated with economic stability (Davenport and Anderson 2005). These differences in perceptions can be attributed to how people consider impacts, positive or negative, in terms of costs and benefits (Quinn et al. 2018). Jacquet and Stedman, (2013) highlight perceptions of impacts as “better predictors of community change and subsequent behavior than measures of the impacts themselves.” These points and those above reinforce why SoP may provide essential avenues for helping to address sustainability needs and making actionable WEF nexus research and management.

### Study Site

Kern County, located in the southernmost portion of California’s San Joaquin Valley (SJV), bears the cumulative impacts of intense rural industrial development, with industrial agriculture and fossil fuel development being the dominant industries (London et al. 2021). Both industries are of colonial origin and follow a typical historical development trajectory towards industrialization, making Kern County an essential case for WEF nexus and sustainability research. Due to its intense rural industrial development and dependence on the production and export of fossil fuel and agriculture for economic growth, Kern County has been characterized as having a resource curse as well as being a fossil fuel and environmental sacrifice zone (Michieka and Gearhart III 2018; Chandrasekaran 2021). The interactions of these industries with water within the context of never-ending economic growth provide a bleak outlook for sustainability. What is more, being a Mediterranean climate, water resources of the county are quickly diminishing due to the demands of these industries, each of which is notorious for unsustainable water consumption, as well as urbanization, population influx, and the impacts of climate change (Almaliki et al. 2022; Keenan and Krannich 1997).

Industrial agriculture and fossil fuel development have deep roots in Kern County. Branded as the engine of the US due to its role in growing the domestic roots of the industry, fossil fuel production for profit in Kern County began in the 1860s (Trout et al. 2018). By 1923, the Midway-Sunset oil field produced a quarter of the global oil supply. It remains one of the top production sites in the US and is the largest oil field in California. Known as California’s fossil fuel capital, Kern County produces about 367,000 barrels of oil per day, provides 70% and 18% of the state’s oil and natural gas, respectively, and is the second largest fossil fuel producer by county in the lower 48 and third in the US providing 5% of US and 1% of global crude oil supply (Thuot 2014; Mernit 2019). The historical development of Kern County’s agricultural industry mirrors the development of its fossil fuel industry. Fed by colonial-era immigration, agricultural development in the county began during the period of the gold rush, as mining and ranching were the ambitions of colonial pioneers and development tycoons (Jelinek 1999; Arax and Wartzman 2003; Turnerjohn 1981). Rancheros and small farms of the County and the broader San Joaquin Valley became the food supply for mining communities until congressional actions led to broadscale privatization of lands, the concentration of land ownership, and the development of industrial agriculture as we know it today (Jelinek 1999). While Kern County is now a top agricultural producer in the US, with crops exported to 96 countries, it produces the most lucrative crops (i.e., almonds, dairy, grapes, and pistachios) in California (CDFA 2022). Kern County’s agricultural lands receive more than 20 million pounds of pesticides each year, contributing to severe environmental pollution and exposure risk (CA DPR 2021). Risks include cancer and neurological diseases, among others (Balazs et al. 2012; Rabinowitz et al. 2015; Wollin et al. 2020).

Kern County is a poster child of rural WEF nexus development and, thus, an essential place for initiating sustainability transitions. For example, while Kern County is ranked as one of the worst in the US for environmental pollution and has been designated as a disadvantaged community of California due to pollution burden and water inequity, it also ranks in the top 75th percentile among California counties for tap water toxicity closely linked fossil fuel and agricultural industry pollution (OEHHA 2021; London et al. 2021; Huang and London 2012; Balazs et al. 2012). A case in point is Kern County’s ranking in the top 68th percentile for 1,2,3-trichlorpropane (TCP) concentrations in California’s tap water, with some census tracts ranking in the top 90th percentile for this carcinogen (OEHHA 2021). 1,2,3-TCP, made by Shell Oil and Dow Chemical, was an ingredient in soil fumigants used in agriculture in California from the 1950s–1980s (Burow et al. 2019; Hauptman and Naughton 2021). Now outlawed, several counties and other organizations are suing Shell and Dow Chemical for the presence of this dangerous carcinogen in local water resources (Burow et al. 2019; Hauptman and Naughton 2021). While peak oil is forecasted to be by 2030 and California aims to be carbon neutral by 2045, the county and state continue to approve new oil and gas development permits (EIA 2023; GOPR 2023; Consumer Watchdog 2023). 35% of the county’s population lives within one mile of an oil or gas well, with nearly half considered vulnerable populations (Rotkin-Ellman 2014). These at-risk communities, especially those working in agriculture, are shouldering the burden due to possible exposure to air pollution, pesticides, and drinking water contamination (Rotkin-Ellman 2014; Perkins and Sze 2011). Cancer is the second leading cause of death in Kern County, and asthma rates are twice that of the state (Constantine and Jonah 2017; CDC 2020).

## Methods

The methods of this research draw heavily from Jacquet and Stedman (2013), who investigated the perceived impacts of wind vs. fossil fuel development projects in addition to place meaning and place attachment as drivers for the perception of impacts. Citing the expansion of energy development projects in rural areas of the US, Jacquet and Stedman (2013) note the importance of understanding why and how residents perceive negative vs positive impacts considering the need to shift towards renewable energy. A primary research objective was to compare perceptions of social, economic, and environmental impacts between the two energy industries, considering the development of each industry may increasingly be in proximity to each other. For example, while research has shown that residents perceive industrial-scale energy development projects as positive for the economy, with social and environmental impacts as negative, other research has shown that residents generally view the environmental impacts of energy development projects as being less important than economic and social concerns (Thompson and Blevins 1983; Jacquet and Stedman 2013). Other findings suggest that while perceptions of negative social impacts include decreases in community connectivity and loss of trust in industrial and environmental regulators, environmental impacts include wildlife habitat destruction, loss of access to environmental amenities, and esthetic disturbances (Mayer 2016; Anderson and Theodori 2009). These place values are important considering they may provide roadblocks to gaining support for environmental protection measures if such measures are viewed as being a threat to the economy.

### Survey Sample and Design

The survey was designed to gauge the perceived impacts of the WEF nexus industries of fossil fuel development and industrial agriculture, place meaning and place attachment as possible drivers for perceptions, and comparison of the perceived impacts between the two industries (Jacquet and Stedman 2013). Duplicate Likert-scale survey questions about industrial agriculture and fossil fuel development impacts were created based on environmental, community, personal, and economic impact constructs. These constructs represent the WEF nexus as a socio-environmental system inclusive of the economy and community as well as the scale of the individual. For each of the 21 variables, the survey asked respondents how each industry has impacted certain aspects of the region where they live by marking one of five boxes for each variable: “very negative,” “negative,” “neutral,” “positive,” “very positive,” whereby negative = damaged/gotten worse, positive = improved/gotten better. Place meaning was gauged by asking respondents to what degree they agree (strongly disagree, disagree, neutral, agree, strongly agree) with statements about the environment, community, and sustainability concerns in Kern County. Place attachment was gauged using the same 5-point Likert scale asking respondents to what degree they agree with four statements (I am deeply connected to this place, I would not want to live anywhere else, I stay here for job security, my job is connected to the land) in addition to Boolean residency status questions. Demographic questions were also included in the survey, considering past research that has shown such variables to be drivers of concern for perception of environmental risks (Jacquet and Stedman 2013). A final question on the survey was open-ended and asked residents to provide any additional information they felt should be addressed regarding the impacts of the fossil fuel industry and/or industrial agriculture on Kern County’s water.

**Fig. 1:**
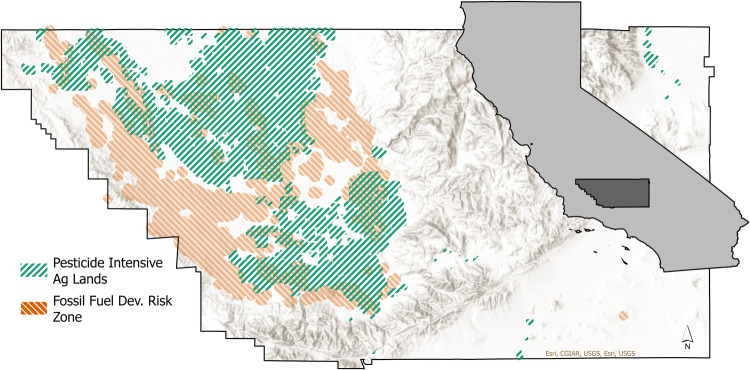
Surveys were distributed to residents who live in the pollution exposure risk zones of the WEF nexus of Kern County. Map by author

Surveys were mailed to residents of Kern County in areas with high concentrations of oil and gas development wells and industrial agriculture (Fig. 1). Survey distribution areas were delineated using a distance-based approach to mapping pollution exposure risk (Mennis 2003; Mennis and Heckert 2018; Haggerty et al. 2019). Research has shown that drinking water wells located <1 km from oil and gas development activities are likely to become contaminated and that individuals living within 2 km of oil and gas development wells may experience adverse health impacts from exposure to related chemicals in water (Rabinowitz et al. 2015; Meng 2015; Wollin et al. 2020). Open-source spatial data of California oil and gas development wells were imported into ArcGIS Pro (V 3.1.0) (CA Department of Conservation 2023). Buffer analysis was used to create a risk buffer of 2 km around active and idle wells. The dissolve tool was used to merge buffers that overlapped to create fossil fuel development risk buffer zones. Research has shown that, due to soil properties, the valley portion of Kern County has a high probability of pesticide contamination in groundwater (Teso et al. 1996). California’s Department of Pesticide Regulation open-source GIS data was used to create a risk buffer of 0.1 km around Kern County agricultural lands that receive the highest applications of pesticides (APC 2019; CA DPR 2021). The overlapping buffer boundaries were dissolved to create an industrial agriculture risk zone. A previous study (Weeks 2023) validated the risk zones by comparing WEF nexus industry-related chemicals in tap water inside, between, and outside of the risk zones. Results showed that, while several chemicals related to WEF nexus industries throughout the valley portion of the county far exceed public health goal safety thresholds, levels were significantly higher within the risk zones and even greater in areas where risk zones overlap (Table 1).

**Table 1:** Average levels of WEF nexus industry-related chemicals in tap water for each risk zone, outside of the risk zones, and the public health goal safety threshold for each chemical

Chemical	Average level in tap water - industrial agriculture risk Zone	Average level in tap water - fossil fuel development risk zone	Average level in tap water - combination risk zone	Average level in tap water - outside of risk zones	CA public health goal safety threshold
TTHMs (ppb)	4.4	27.3	12.9	7.2	0.015
HAA9 (ppb)	2.4	27.9	4.9	0	0.06
Chromium-6 (ppb)	4.17	0.64	1.02	0	0.02
1,2,3-TCP (ppb)	0.014	0.0003	0.038	0	0.0007
DBCP (ppb)	0.01	0.00003	0.004	0	0.0017
Arsenic (ppb)	3.7	1.38	8.8	4.08	0.004
Nitrate (ppm)	5	1.9	2.3	1.2	0.14

Weeks 2023.

USPS Every Door Direct Mail (EDDM) was used for the anonymous distribution of and response to the survey. Mail routes entirely within the risk zones were chosen for survey distribution. Each survey contained a pre-paid USPS business reply mail envelope for survey responses to be sent to a USPS P.O. Box. Surveys were sent to a wide variety of zip codes to decrease bias in the event of a low response rate, with an equal number of surveys sent to the fossil fuel and industrial agriculture risk zones to obtain a representative proportion of responses from populations working or residing near those industries. Further, two versions of the survey (English and Spanish) were sent to the agricultural areas considering the percentage of the population in those areas that are non-English speaking farm working communities. Two thousand five hundred surveys were sent with a response rate of 10.2% (246 English and 10 Spanish surveys returned). The response rate was much higher for the fossil fuel industry risk zone (16%) versus the industrial agriculture risk zone (4%). Even with the more significant response rate from the fossil fuel development risk zone, 18% of the respondents were agricultural workers, while 13% worked in the fossil fuel industry. The survey sample provided answers from all categories of demographics from Kern County. Most respondents were white (69.8%), followed by Latinx (16.5%), Native American (9.4%), Asian/Asian American (2.7%), and African American (1.2%). 58.8% of the respondents were female and 35.7% were male. Limitations of the survey include the low response rate from Latinx communities, considering they represent about 50% of the county, and the low response rate.

### Analysis and Results

While the analysis is primarily quantitative, as shown below, qualitative analysis of the open-ended question on the survey was used to identify themes, which were utilized for complementary insights into the perceived impacts, place meaning, and place attachment. Likert-scale survey data were imported into SPSS (V. 29.0). Factor analysis, descriptive statistics, and bivariate analyses were used to evaluate the survey responses (N = 256). While factor analysis was used to examine perceived impact groupings per industry compared to the original survey categories, descriptive statistics were used to gauge perceived impacts, place attachment, and place meaning. Bivariate regression was used to test correlations between SoP variables and perceived impacts to obtain insights into place meaning and place attachment as drivers for perceived impacts (Jacquet and Stedman 2013). The reliability of the questionnaire was confirmed by calculating the Cronbach’s alpha for each category of questions with perceived impacts survey items scoring 0.96, place meaning items scoring 0.84, and place attachment items scoring 0.741. The open-ended survey responses (N = 100) were imported into NVIVO (V. 14.23.2). Auto-coded themes were agriculture, air, fossil fuel, industry, oil, quality, and water. Comments related to these themes provided complementary insights into place meaning and place attachment as related to the perceptions of the impacts of WEF nexus industries as those industries intersect with the environment, the community, the economy, and the individuals taking part in the survey.

### Perceived Impacts

Factor analysis was used to examine the perceived impacts responses for each industry. This provided for the isolation of constructs and concepts by regrouping variables into variable cluster sets referred to as “factors” while also providing for comparison of the factor constructs with the questionnaire categories (Yong and Pearce 2013). The factor analysis used principal components extraction based on Eigenvalues greater than 1 with varimax rotation for each industry (Jacquet and Stedman 2013). The Keiser-Meier-Olkin measure for sampling adequacy was 0.921 for industrial agriculture and 0.918 for fossil fuel development, while Bartlett’s test of sphericity provided a significance (P-value) of < 0.001 for the survey items for each industry, thus indicating the data’s adequacy for factor analysis and that the variables were statistically significant (Yong and Pearce 2013). The factor analysis for each industry provided similar outcomes with factor constructs mirroring the questionnaire categories. For example, while there were two resulting factors for the perceived impacts of industrial agriculture, there were three factors for the fossil fuel industry. The two factors for the perceived impacts of industrial agriculture loaded as per “environmental and personal impacts” and “social and economic impacts.” The three factors for the perceived impacts of the fossil fuel industry loaded as per “environmental and personal impacts,” “community impacts,” and “economic impacts.”

Descriptive statistics (Table 2) show that, while participants were somewhat neutral in their perceptions of the impacts of both industries for many variables, residents had strong perceptions of impacts, negative and positive, for key variables which are bolded in the table. Industrial agriculture was perceived as having the most significant negative impacts.

**Table 2:** Percentages of the perceived impacts of industrial agriculture vs fossil fuel development in pollution exposure risk zones of Kern County, CA

Impacts	Industrial Agriculture		Fossil Fuel Development	
	negative	positive	negative	positive
Environmental				
water quality	39.7	15.8	38.6	19.1
Tap water quality	**46.3**	20.8	40.6	21.1
air quality	**62.3**	12.9	**53.3**	16.5
wildlife habitat	36.2	26.8	38.1	22.8
Access to water	25.6	34.1	27	27.8
Scenic beauty	28.3	36.4	36.6	23.7
Personal Impacts				
Resident health	39.3	23.8	39.9	22.4
Pollution exposure risk	**55.7**	41.8	**52.7**	43.2
Quality of life	30.9	**58.5**	35.3	31.6
Community impacts				
Trust in local government	**51.8**	12.5	**52.3**	8.7
Community connectivity	32.9	25.6	35.3	21.5
Pride in community	28.7	35.2	31.2	30.8
Inclusion in planning	39.7	15.4	41.1	15.3
Economic impacts				
Property values	31.8	30.1	34.9	29.9
Job market	32.9	34.5	34.6	35
Economic health	38.9	29.1	35.2	32.8

Likert scale questions asked how WEF nexus industries have imposed environmental, personal, community, and economic impacts by marking one of five boxes for each variable: “very negative,” “negative,” “neutral,” “positive,” “very positive,” whereby negative = damaged/gotten worse, positive = improved/gotten better

The bolded numbers represent strong perceptions, negative or positive.

#### Environmental Impacts

While the environmental impacts from WEF nexus industries were generally perceived as negative, slightly more so for industrial agriculture than fossil fuel, some impacts were perceived as positive. Air and water quality was perceived as being negatively impacted by both industries but more by industrial agriculture (industrial agriculture 62.3%, fossil fuel development 53.3%). Water quality was perceived as being negatively impacted but less so than air quality. This may be attributed to a lack of awareness of water pollution versus the ability to see and feel the effects, such as allergies or asthma, of air pollution on a regular basis. While perceptions of the impacts of industrial agriculture on scenic beauty were more positive (36.4) than negative (28.3), the perception of the impacts of fossil fuel development for that variable were more negative (36.6) than positive (23.7). It is important to note the percentage of neutral responses for the perceptions of environmental impacts which were generally 30% for half of these variables.

#### Personal Impacts

The perceptions of personal impacts were among the least neutral variables, with responses showing a sharp divide in perceptions. For example, while both industries were perceived as having negative impacts on pollution exposure risk, slightly more so for industrial agriculture (55.7%) than fossil fuel (52.7%), nearly an equal percentage of participants perceived WEF nexus industries as having a positive impact on pollution exposure risk. Meanwhile, quality of life was perceived as being positively impacted by industrial agriculture (58.5%), with perceptions being slightly more negative than positive for fossil fuel development for that variable. In consideration that 50% of the population of Kern County are Latinx, a comparison of responses regarding environmental and personal impacts perceived by these survey participants versus the rest of the participants could provide insights into potential environmental justice burdens. Results were similar between the two populations except for a 4% increase for pollution exposure risk from industrial agriculture for Latinx survey participants.

#### Community and Economic Impacts

The perceptions of community and economic impacts were mixed. Trust in local government was strongly perceived as being negatively impacted by both industries but more so by fossil fuel development. Similarly, participants perceived inclusion in planning as being negatively impacted by both industries, slightly more so by fossil fuel development. Alternately, pride in community was perceived as being positively impacted by industrial agriculture while being more negatively impacted than positive for fossil fuel development. Perceptions of the impacts of both industries on the economic variables of property values, job market, and economic health were generally evenly distributed between negative, neutral, and positive. Economic health and property values were perceived as being more negatively impacted than positive.

## Sense of Place and Correlations with Perceptions of Impacts

### Place meaning

#### Environment as Restorative

While participants disagreed that Kern County’s environment is healthy (60%) and that it is a good place to get away (42%), there was a greater percentage of responses that agreed that Kern County has great outdoor recreation (44.5%) and outstanding natural beauty (45.4) than those who disagreed (25.6% and 22.9% respectively) (Table 3). This contradiction may be explained by the perceived positive impact of industrial agriculture on scenic beauty considering esthetics as part of place meaning (Berleant 1992). Bivariate correlation analysis between the variables “Kern County has outstanding natural beauty” and the perceived impacts of industrial agriculture variable for scenic beauty was statistically significant (<0.001) with a positive Spearman’s correlation coefficient (0.619), indicating place meaning as related to esthetics is a driver for the perceived positive impact. Alternately, fossil fuel development was more greatly perceived as having a negative impact on scenic beauty and positively correlated with the place meaning variable of Kern County having outstanding natural beauty. Thus, as fossil fuel development expands, the greater the negative impacts perceived on the esthetic nature of Kern County.

**Table 3:** Percentages for survey responses to Likert-scale questions about place meaning

Place meaning categories and variables	Disagree	Agree
Environment as restorative		
The environment is healthy	**59.9**	19.7
Kern County is a good place to get away	41.5	22.9
Kern County has great outdoor recreation	25.6	44.5
Kern County has outstanding natural beauty	22.9	45.4
Community		
The community is close-knit	29.9	30.7
The community is very friendly	22.1	45.6
Economy		
My job is connected to the land	44.8	25.4
Threatened		
Water quality is in decline	20.1	**53**
The economy is in decline	13.4	**68.8**
I am worried about sustainability in Kern County	13.8	**60.9**

The bolded numbers represent strong perceptions, negative or positive.

### Community

While responses were divided about Kern County’s community being close-knit, there were more responses that agreed the community is very friendly (45.6%) than those of who disagreed. (22.1%). While community connectivity is important for community planning and management, other factors outside of this research are most likely having an impact on these variables, such as urban growth and migration of individuals from urban areas to more affordable regions such as Kern County. Community connectivity is discussed further in the following sections.

### Economy

While there were two place meaning variables related to the economy, one is in the threatened category and the other in this category sought to ascertain if residents in the WEF nexus industry pollution exposure zones felt their job was connected to the land. While 44.8% of respondents disagreed, 25.4% agreed. There was a significant positive correlation between this place meaning variable and perceptions of the impacts of WEF nexus industries on water quality, air quality, and pollution exposure risk. This means the more individuals see their job as being connected to the land, the greater their perceptions of impacts, which aligns with past SoP research related to this variable (Cross et al. 2011).

### Threatened

The “threatened” category of place meaning provided the least neutral responses. Respondents agreed they are worried about sustainability (60.9%) in Kern County and that the economy is in decline (68.8%) while also agreeing that water quality is in decline (53%). Similar to Jacquet and Stedman’s (2013) findings, respondents who agreed with variables in the “threatened” category of place meaning had greater perceptions of WEF nexus industry impacts. While there was a significant correlation (<0.001) between the place meaning variable of the declining economy and the perceived impacts of industrial agriculture on water quality, there was not for fossil fuel development. Further, the Spearman’s correlation coefficient for perceived impacts of industrial agriculture on water quality with the economy being in decline was -0.217, thus indicating a negative relationship between the variables.

The open-ended question at the end of the survey provided more profound insights into place meaning in Kern County’s WEF nexus, complementary to the above findings, while also showing a deep divide in place meanings related to WEF nexus industries. For example, comments related to the auto-coded theme of industry showed that, while many respondents view Kern County as being WEF nexus industries (i.e., “Kern County is fossil fuel and industrial agriculture”), others view WEF nexus industries as turning Kern County into an export economy that is destroying the environment (“the almond industry takes our water and ships its products overseas”). This shows that industrial development is an important driver of place meaning and a source of contested place meanings. Many comments by respondents reinforced the correlation between their disagreement with the variable “the environment is healthy” and the perceptions of WEF nexus industry impacts on the environment and human health. For example, many respondents commented on the negative impacts of both industries on air and water quality while associating those impacts with their personal experiences of degraded qualities of the environment (“As a resident of Oildale I have to avoid the outside air” “We are a top producer of agriculture in the nation yet we have the worst air quality and health conditions” “The air quality here is horrible because I step out in the morning to that awful stench of gasoline toxins” “Rural areas around Kern County are often discarded regarding water quality and agricultural practices”).

### Place attachment

The median for the place attachment Likert scale survey items responses (3.0) indicates that place attachment is weak among the respondents (Table 4). While respondents agreed most (43.5%) with the statement “I am deeply connected to this place,” they disagreed more (58.1%) with the statement “I would not want to live anywhere else.” Furthermore, 44.3% disagreed that their job is connected to the land. This is an interesting outcome, considering that 31% of the survey participants work in fossil fuel development or agriculture. Bivariate correlation analysis indicates that place attachment may be an important basis, or driver, for perceptions of the impacts of industrial development. For example, recall that responses indicated greater perceptions of the negative impacts of industrial agriculture on water quality than fossil fuel development. The correlation between the variable “I am deeply connected to this place” and the perceived impacts of WEF nexus industries in water quality was statistically significant (<0.001) for each industry but stronger for industrial agriculture (r = 0.311** for fossil fuel and 0.375** for industrial agriculture). Further, while the correlations between place attachment variables and perceived impacts of WEF nexus industries on environmental quality were statistically significant, the correlations were positive, thus indicating that as place attachment increases, so do the perceptions of impacts. Relative are insights that can be drawn from residency status as related to related to place attachment. Whereas lifetime residency and place attachment were weakly correlated (r = 0.189**), the correlation between year-round residency and place attachment was not statistically significant (P = 0.544). While this backs SoP research that has shown that place attachment develops over time, lifetime residents being 50% of survey respondents should have resulted in greater agreement with place attachment survey variables. This discrepancy may be related to place meaning or place attachment becoming subsumed into industrial development, which is considered in the discussion section.

**Table 4:** Percentages of survey responses to the Likert-scale questionnaire items on place attachment

Place attachment	Disagreed	Agreed
I am deeply connected to this place	29	43.5
I would not want to live anywhere else	58.1	18
I stay here for job security	35.3	30.2
My job is connected to the land	44.3	25.1

Like place meaning, survey responses to the final open-ended survey question provided complementary insights into place attachment. For example, the auto-coded industry theme provided more profound insights into place connection whereby WEF nexus industries are experienced as an integral part of the local social system, providing multi-faceted stability to local livelihoods. Responses show this to be especially true for the fossil fuel industry as participants highlighted its important contributions to the economy, schools, community outreach, and police. This is an important finding because proposed changes to that basis for stability, such as decarbonization, may be viewed as a threat. Exemplary comments include, “the oil industry provides quality, decent paying jobs in this community that wouldn’t exist without the oil industry,” “Kern County prospers from the oil industry through land taxes and permit fees that help pay for police and schools.” Examples of this dynamic were also present in participant comments about the need to deregulate the fossil fuel industry, such as the “fossil fuel industry lacks jobs because of state regulation” and “open up the oil industry so people can get back to work.” There were also clear concerns for the closure of the fossil fuel industry in comments such as, “leave the oil industry alone” and “The fossil fuel industry is an important part of Kern County and should not be shut down.”

## Discussion

This research found that aspects of place meaning and place attachment are drivers of perception of the impacts of WEF nexus industries as shown with similar research on SoP (Jacquet and Stedman 2013), though it also found that aspects of place meaning and place attachment are drivers of concern for changes in the local industry. This discussion focuses on the latter first, then explores the former in consideration of ways to increase sustainability management and transitions policy support in the WEF nexus. Two key findings of this research were that WEF nexus industries have shaped place meaning for Kern County residents and that place attachment is rooted to these industries, particularly fossil fuel, as livelihoods through monetary support for local institutions such as schools and police are supported. Place meaning and attachment being formed over time through personal and social experience as well as livelihood stability provided by WEF nexus industries thus undergirds the drive for concern for changes. This is not a surprising finding considering the length of time these industries have been part of the fabric of Kern County’s socio-environmental system. However, it is an important finding for sustainability management policy support in the WEF nexus.

As theorized by Tuan (1975), place meaning develops through lived experiences. However, what about dominant constructs of place meaning and attachment – those of community, culture, and political-economic relevance? Stedman (2016) briefly dives into the problem of systemic power influencing the construction of place meaning, pointing to the works of Foucault (2009) and Gramsci (1971), who explain that institutional and systemic power influences the normalcy of meanings, behavior, and even common sense. This line of thought related to SoP has been explored deeply by David Harvey (1993; 2018), who, using Marx’s theory of alienation, explains how universal alienation materializes as capital accumulation becomes the goal of life under the current political-economic ontology. Harvey (1993) quotes Relph (1976), who warned that place is being rendered placeless by “organizational power and depth of penetration of the market” in the logic of individuals. Similarly, Bell and York (2010) exemplify the treadmill of production as being reinforced by the manipulation of place attachment by the coal industry who constructed an ideology of dependency and economic identity. These insights may explain the weak strength of place attachment demonstrated by survey participants and the general neutrality for more than half of the perceived impacts variables, even in the face of severe environmental degradation in Kern County. These findings present a deeper problem that needs to be addressed at the personal level of the WEF nexus as a system—to strengthen place attachment and build upon aspects of place meaning to cultivate support for sustainability transition policies such as decarbonization and regenerative agriculture.

Confronted with alienation, how might SoP in Kern County’s WEF nexus be utilized, improved, or recovered to achieve sustainability management? One pathway may be provided by focusing on the restorative quality of nature as a factor of place meaning considering this research found it to be an important driver for perceptions of the negative impacts of WEF nexus industries. Drawing from Stedman (2002), who explained that humans are willing to fight for places that are more central to their identities and perceive as being in less-than-optimal conditions, nurturing place identity and meaning aligned with a healthy environment would be an important path forward. In recognition of this need, some promote SoP as a cultural ecosystem service to develop place meanings and connections, personal and social systemic, between humans and local ecosystems. For example, personal and group involvement in ecosystem restoration activities has been shown to build place connection and identity, thus nurturing support for conservation policy (Lokhorst et al. 2014; Hausmann et al. 2016). There are seemingly endless opportunities for ecosystem restoration in the heavily industrialized ecosystems of Kern County. Rivers have often been the focal point for such activities (Quinn et al. 2018), as well as the development of small-scale agriculture as a win-win discourse of conservation based on place meaning and place attachment (Masterson et al. 2019).

There was a statistically significant association between the variable “the economy is in decline” of the threatened category of place meaning and the environment/restorative category variable “Kern County has outstanding natural beauty” thus backing this line of thought and proposed action. It would be advantageous then to provide avenues to demonstrate and build upon the restorative nature of the environment as integral to the WEF nexus and, thus, the long-term sustainability of the economy. For example, 44% of respondents agree that Kern County has outstanding natural beauty and great outdoor recreation, thus indicating these aspects of place meaning may be point of pride. Past research has shown that pride in a place as one that highly values its ecosystems can strengthen place attachment (Marshall et al. 2019). Relatively, while survey participants perceived industrial agriculture as having a positive impact on scenic beauty, they perceived that industry as having the most negative impacts on the environment and pollution exposure risk. Place meaning related to the beauty of agriculture and place-based pride could be enhanced by transitioning to agricultural practices that build ecosystem resilience.

The threatened category of place meaning survey items were most agreed with among all SoP survey variables, with nearly 70% viewing Kern County’s economy as being in decline and 60% being worried about sustainability. There needs to be a greater effort to assure Kern County residents that sustainability transitions in the WEF nexus, such as decarbonization and regenerative agriculture, will greatly benefit the community and economy instead of being a threat. Relative to the perception of threat to the economy, responses showed that residents perceive WEF nexus industries as having a negative impact on trust in the government. This critical finding indicates a need for greater grass-roots involvement in planning and decision-making processes (Armitage et al. 2007). For example, Johnson and Rickard (2022) found that seeing community change as positive was increased using a cooperative management approach. In terms of the fossil fuel industry, just transitions are needed to ensure that renewable energy jobs pay as well (or better) as those of the fossil fuel industry and that those working in fossil fuel get training and job security during the transition (Healy and Barry 2017). Moreover, considering place attachment was found to be a driver of concern for threats to the fossil fuel industry due to monetary support (land taxes and permit fees) for social institutions from that industry, support for Kern’s social systems needs to be enhanced from its renewable energy sector which generates far more renewable energy than any other county in California (Zhang et al. 2022).

Finally, this research found conflicting views about the impacts of WEF nexus industries, thus representing contested visions of sustainability in Kern County’s WEF nexus. Chapin III and Knapp (2015) suggest that “stewardship is best fostered by transparent and respectful dialog to identify shared values and concerns and negotiate areas of disagreement.” Providing arenas (workshops, community forums, planning meetings) for such activities in Kern County could provide opportunities for discourse among residents to increase awareness of shared concerns for sustainability transitions, environmental pollution related to WEF nexus industries, and shared values related to place meaning and place attachment such as those found in this research. Further, this research found that the survey participants disagree the community is close-knit, which could be shifted through such venues for dialog. Such venues could also build social networks and ultimately strengthen place attachment.

## Conclusion

This research demonstrates the importance of social science and the relevance of SoP in WEF nexus research and management. Rooting WEF nexus research to the local social dimension as bound to the broader socio-environmental system provided important insights into local perceptions as well as place meaning and place attachment as drivers for perceived impacts and concerns for community change. An important finding of Kern’s WEF nexus is that, due to long-term industrialization, WEF nexus industries have shaped place meaning and that WEF nexus industries, particularly fossil fuel, are experienced as being an integral part of the local social system providing multi-faceted stability to local livelihoods. Place meaning and attachment are formed over time through personal and social experience, as well as livelihood stability provided by nexus industries, which are drivers for concern for changes in WEF nexus industries. These concerns need to be relieved and trust in government and policy built, which can be achieved via cooperative management and arenas for sharing knowledge and concerns. Just transitions are needed also to alleviate concerns for community change. Weak place attachment and related alienation, or placelessness, is an important outcome in the rural industrialized WEF nexus that needs tending and mending to increase support for sustainability transitions, particularly for decarbonization. Nurturing place identity and meaning as being aligned with a healthy environment provides an important path forward, which can be aided through personal and group activities to build pride in healthy ecosystems in Kern County areas impacted by WEF nexus industries.

Critical actions for aligning constructs of SoP with sustainability management in the rural industrialized WEF nexus materialized from this research. To gain support for sustainability policy and transitions, the environment as being restorative as a key factor of place meaning needs to be developed. A key avenue to do this is through ecosystem restoration projects that involve the community and individuals. These projects should include river restoration, the implementation of small-scale and regenerative agriculture, and the remediation of fossil fuel development areas. In addition to making environmental amenities a greater aspect of the economy, these activities will alleviate the threatened factor of place meaning. Cooperative management and increased monetary support for local sectors of the community (schools, police) from the renewable energy sector are also needed. Cooperative management will provide arenas for discourse between individuals with contested visions of sustainability and building trust for government and policy. These WEF nexus management foci will also help to strengthen place attachment and repair environmental alienation.

### Supplementary information

Survey
