# Correction: Sense of Place and Perceived Impacts in the Rural Industrialized Nexus: Insights for Sustainability Pathways

**DOI:** 10.1007/s00267-024-01985-3

**Published:** 2024-05-23

**Authors:** Deseret Weeks, Jeffrey Jenkins

**Affiliations:** Department of Management of Complex Systems, University of California, Merced, CA USA

Correction to: Environmental Management (2024)

10.1007/s00267-024-01969-3

In this article under the “Introduction” section in the fourth paragraph the sentence “This conversion is expected to be accompanied by a 2.4–2.7-fold increase in nitrogen and phosphorus-driven eutrophication and a similar increase in the use of pesticides (Tilman et al. 2001).” has been corrected to “This conversion is expected to be accompanied by a 2^4^–2.^7^-fold increase in nitrogen and phosphorus-driven eutrophication and a similar increase in the use of pesticides (Tilman et al. 2001)”.

The original article has been corrected.

