# Supplementary material for: Sense of Place and Perceived Impacts in the Rural Industrialized Nexus: Insights for Sustainability Pathways
Source: Environ Manage. 2024 Apr 5;73(6):1215–29. doi: 10.1007/s00267-024-01969-3 (PMC11136804; doi:10.1007/s00267-024-01969-3)
Supplement: Supplementary file 1 — Survey [file 267_2024_1969_MOESM1_ESM.pdf]

Kern County Residents Matter!

You are being asked to participate in a research study. This research aims to learn about the experiences, and points of view, of individuals living near fossil fuel development and/or industrial agriculture as these industries intersect with water and the communities of Kern County.

Benefits: Your responses to the survey questions will provide important insights into Kern County resident experiences which may ultimately influence local planning and management decisions.

Procedures:

- Please provide responses to all questions of the survey and any additional insights you feel are important in the space provided. The questionnaire should take less than 10 minutes to complete. Your responses are completely anonymous.
- **You are asked to return the completed survey using the included postage-paid envelope.**

Your completion of this survey implies your consent to take part in this research.

Thank you so much for taking the time to read, complete, and mail this survey.

To what extent do you agree or disagree with the following statements about Kern County?

	Strongly disagree	*Disagree*	*Neutral*	*Agree*	*Strongly agree*
The environment is healthy	☐	☐	☐	☐	☐
It is a good place to get away	☐	☐	☐	☐	☐
It has outstanding natural beauty	☐	☐	☐	☐	☐
It has great outdoor recreation	☐	☐	☐	☐	☐
The community is very friendly	☐	☐	☐	☐	☐
The community is close-knit	☐	☐	☐	☐	☐
I stay here for job security	☐	☐	☐	☐	☐
I would not want to live anywhere else	☐	☐	☐	☐	☐
I am deeply connected to this place	☐	☐	☐	☐	☐
My job is connected to the land	☐	☐	☐	☐	☐
Water quality is in decline	☐	☐	☐	☐	☐
The economy is in decline	☐	☐	☐	☐	☐
I am worried about sustainability in Kern County	☐	☐	☐	☐	☐

Please identify how agricultural development has impacted Kern County (Negative = damaged/gotten worse, Positive = improved/gotten better)

	Very Negative	Negative	Neutral	Positive	Very positive
Water quality	☐	☐	☐	☐	☐
Air quality	☐	☐	☐	☐	☐
Wildlife habitat	☐	☐	☐	☐	☐
Access to water	☐	☐	☐	☐	☐
Scenic beauty	☐	☐	☐	☐	☐
Trust in local government	☐	☐	☐	☐	☐
Community connectivity	☐	☐	☐	☐	☐
Pride in community	☐	☐	☐	☐	☐
Inclusion in planning	☐	☐	☐	☐	☐
Resident health	☐	☐	☐	☐	☐
Pollution exposure risk	☐	☐	☐	☐	☐
Tap water quality	☐	☐	☐	☐	☐
Quality of life	☐	☐	☐	☐	☐
Property values	☐	☐	☐	☐	☐
Job market	☐	☐	☐	☐	☐
Economic health	☐	☐	☐	☐	☐

Please identify how fossil fuel development has impacted Kern County (Negative = damaged/ gotten worse, Positive = improved/gotten better)

	Very Negative	Negative	Neutral	Positive	Very positive
Water quality	☐	☐	☐	☐	☐
Air quality	☐	☐	☐	☐	☐
Wildlife habitat	☐	☐	☐	☐	☐
Access to water	☐	☐	☐	☐	☐
Scenic beauty	☐	☐	☐	☐	☐
Trust in local government	☐	☐	☐	☐	☐
Community connectivity	☐	☐	☐	☐	☐
Pride in community	☐	☐	☐	☐	☐
Inclusion in planning	☐	☐	☐	☐	☐
Resident health	☐	☐	☐	☐	☐
Pollution exposure risk	☐	☐	☐	☐	☐
Tap water quality	☐	☐	☐	☐	☐
Quality of life	☐	☐	☐	☐	☐
Property values	☐	☐	☐	☐	☐
Job market	☐	☐	☐	☐	☐
Economic health	☐	☐	☐	☐	☐

Please answer the following questions by checking the box for "yes" or "no"

	Yes	No
I live in Kern County year-round	☐	☐
I have lived in Kern County my whole life	☐	☐
I am female	☐	☐
I am male	☐	☐
I am Native American	☐	☐
I am African American	☐	☐
I am Latino/Latina	☐	☐
I am Asian or Asian/American	☐	☐
I am white or of European descent	☐	☐
I work in agriculture	☐	☐
I work in the fossil fuel industry	☐	☐
I have a college degree	☐	☐
My highest level of education is high school	☐	☐

Please provide any additional information or insights on what you feel should be addressed regarding the impacts of the fossil fuel industry and/or industrial agriculture on Kern County's water.
